# Neurological Manifestations of Dengue Infection

**DOI:** 10.3389/fcimb.2017.00449

**Published:** 2017-10-25

**Authors:** Guo-Hong Li, Zhi-Jie Ning, Yi-Ming Liu, Xiao-Hong Li

**Affiliations:** ^1^Department of Neurology, Jinan Central Hospital Affiliated to Shandong University, Jinan, China; ^2^Jinan Infectious Diseases Hospital, Jinan, China; ^3^Department of Neurology, Qilu Hospital, Shandong University, Jinan, China

**Keywords:** dengue, neurological complications, neuropathogenesis, in children, manifestations, treatment, prevention

## Abstract

Dengue counts among the most commonly encountered arboviral diseases, representing the fastest spreading tropical illness in the world. It is prevalent in 128 countries, and each year >2.5 billion people are at risk of dengue virus infection worldwide. Neurological signs of dengue infection are increasingly reported. In this review, the main neurological complications of dengue virus infection, such as central nervous system (CNS), peripheral nervous system, and ophthalmic complications were discussed according to clinical features, treatment and possible pathogenesis. In addition, neurological complications in children were assessed due to their atypical clinical features. Finally, dengue infection and Japanese encephalitis were compared for pathogenesis and main clinical manifestations.

## Introduction

Dengue counts among the most common arboviral illnesses, representing the fastest spreading tropical disease in the world (Lundberg, 1957). It is considered the second leading cause of acute febrile disease in travelers (Freedman et al., 2006). Four different serotypes (DENV-1,−2,−3, and−4) (Guzman et al., 2010) cause dengue fever, with various infectious outcomes (asymptomatic to severe hemorrhagic fever).

Dengue is prevalent in 128 countries (Brady et al., 2012; Lorenzi et al., 2013; Teixeira et al., 2013), and more than 2.5 billion individuals are in danger each year of contracting dengue virus worldwide. According to some estimates, almost 400 million individuals are infected annually, with ~96 million showing clinical relevance. About 2.5% of all diseased people die (Guzman et al., 2010; Bhatt et al., 2013). In recent years, neurological manifestations of dengue infection have been increasingly reported; however, their precise incidence rates remain undefined.

Neurological signs were first reported in 1976 as atypical symptoms of dengue infection (Sanguansermsri, 1976); their incidence rates varied from 0.5 to 20% in recent years (Murthy, 2010; Carod-Artal et al., 2013; Mamdouh et al., 2013; Sahu et al., 2014; Saini et al., 2017). Neurological manifestations have been reported in 25 countries spanning almost all continents (Qureshi et al., 2012), and involve individuals aged 3 months to 60 years (Qureshi et al., 2012). High body temperature, elevated hematocrit, thrombocytopenia, rash, and liver dysfunction are independent risk factors for neurological complications (Sahu et al., 2014).

Almost 20 years ago, dengue virus neurotropism in the human host was considered an opportunistic characteristic (Ramos et al., 1998). However, more and more evidence strongly supports the notion that the virus is directly neurovirulent (Rosen et al., 1989; Bhoopat et al., 1996; Lum et al., 1996; Miagostovich et al., 1997b; Ramos et al., 1998; Angibaud et al., 2001). Miagostovich et al. detected the dengue virus in the central nervous system (CNS) by assessing viral proteins, ribonucleic acid (RNA), and immunoglobulins (Miagostovich et al., 1997a,b; Araujo et al., 2011; Lima et al., 2011). Salazar et al. (2007) found that the dengue virus is highly neurotropic in *Aedesaegypti* (Bhoopat et al., 1996). The DENV-2 and DENV-3 serotypes are mostly related to neurological complications (Lum et al., 1996; Thisyakorn et al., 1999; Miagostovich et al., 2006; Soares et al., 2010).

## Neuropathogenesis

Neuropathogenesis is likely associated with direct invasion of the CNS by the virus, autoimmune reactions, and metabolic alterations. The dengue virus is considered to be non-neurotropic. However, recent reports associating dengue with neurological complications have changed this view. This virus was described in cerebro-spinal fluid (CSF) more than two decades ago (Lum et al., 1996; Thisyakorn et al., 1999). Chaturvedi et al. demonstrated that the bloodbrain barrier (BBB) is damaged during infection by the dengue virus in experimental animal experiments, indicating viral invasion (Chaturvedi et al., 1991). Meanwhile, immunoreactive neurons, astrocytes, microglia, and endothelial cells were found in cerebral tissues of a fatal case with hemorrhagic dengue fever in 1998 (Ramos et al., 1998). Domingues et al. proposed that dengue virus can actively enter the CNS (Domingues et al., 2008). Data obtained in Vietnam also support direct invasion by dengue virus as pathologically important (Solomon et al., 2000b). Autoimmune reactions and metabolic alterations have been demonstrated in most neurological complications of dengue fever cases (Seet and Lim, 2007; Basu and Chaturvedi, 2008; Jha and Ansari, 2010; Murthy, 2010; Sharma et al., 2011; Verma et al., 2011b; Weeratunga et al., 2014b).

Most neurological manifestations of dengue virus infection have been reported in case reports or short series, and its spectrum is diverse; thus, the classification of neurological manifestations is difficult to apply in practice. The new (2009) World Health Organization (WHO) classification groups dengue infection into three categories, including dengue with no warning signs, disease with warning signs, and severe dengue (WHO, 2009). Different from the traditional system, the revised classification includes CNS involvement as severe dengue. However, neurological complications are not well described and very little is known about these manifestations.

Until 2012, neurological complications of dengue virus infection were classified into three categories based on pathogenesis as proposed by Murthy, Marzia and colleages: (1) metabolic disturbance, e.g., encephalopathy; (2) viral invasion, including encephalitis, meningitis, myositis, and myelitis; (3) autoimmune reactions, including acute disseminated encephalomyelitis, neuromyelitis optica, optic neuritis, myelitis, encephalopathy, and Guillain-Barré syndrome (Murthy, 2010; Puccioni-Sohler et al., 2012). In recent years, Solbrig et al. reported neurological involvements of the CNS and eyes, associated peripheral nervous system (PNS) syndromes, and convalescent or post-dengue immune-mediated syndromes (Solbrig and Perng, 2015; Maurya et al., 2016).

The main neurological complications of dengue virus infection are discussed below.

## Central neurological system complications

CNS complications are diagnosed by assessing anti-DENV immunoglobulin (Ig)M, detecting viral RNA or non-structural protein 1 (NS1)in the CSF, isolating the virus from the CSF, and after excluding other causative agents of viral brain diseases (Sahu et al., 2014; Solbrig and Perng, 2015). Misra et al. found that CNS involvement reflects severer disease with poorer recovery (Misra et al., 2015). Encephalitis and encephalopathy are the most common neurological presentations of dengue infection (World Health Organization, 1997; Pancharoen and Thisyakorn, 2001; Gupta et al., 2013; Table 1).

**Table 1:** Main central neurological system complications associated with dengue infection.

**Complications**	**Main symptoms and signs**	**CSF parameters**	**CT/MRI**	**References**
Encephalitis	Acute signs of cerebral involvement	Normal cell count/Pleocytosis, normal/High level of protein	Normal/Signal changes in involved regions	Solomon et al., 2000b; Soares et al., 2011; Baldaçara et al., 2013; Madi et al., 2014; Mathew et al., 2015; Garg et al., 2017
Encephalopathy	Cognitive disorders, convulsions, mood/personality/behavior disorders	Normal in most cases	Suggestive of extensive involvement of the bilateral cerebellar region, brainstem, and thalami along with peculiar rim enhancement (MRI)	Sumarmo et al., 1983; Nimmanitya et al., 1987; Srivastava et al., 1990, 2013; Hendarto and Hadinegoro, 1992; Thisyakorn and Thisyakorn, 1994; Sistayanarain et al., 1996; Sirivichayakul et al., 2000; Solomon et al., 2000b; Angibaud et al., 2001; Misra et al., 2006; Liou et al., 2008b; Rittmannsberger et al., 2010; Oehler et al., 2012; Baldaçara et al., 2013
Meningitis	Acute onset of fever and symptoms such as headache, vomiting, and/or nuchal rigidity; absence of parenchymal involvement	CSF cell count greater than 5 cells/mm^3^, and negative tests for bacteriological and fungal infections	Cranial CT was normal	Soares et al., 2010; Mamdouh et al., 2013
Stroke				
Ischemic stroke	Focal neurological signs such as hemiparesis, dysarthria, and so on	15 cells (all lymphocytes) with normal protein and sugar levels	Hypodensity on cranial CT	Liou et al., 2008a; Verma et al., 2013
Hemorrhage stroke	Headache, vertigo, vomiting, somnolence, hemiparesis, and dysarthria	Normal/Hemorrhagic CSF if blood escapes into the ventricular system	Hyperdensity on cranial CT	Seet and Lim, 2007; Vargas-Sánchez et al., 2014
Cerebellar syndrome	Bilateral vertical and horizontal nystagmus, dysarthria, bilateral limb, and gait ataxia	Normal	Normal/Cerebellar T2 hyperintense lesions (MRI)	Weeratunga et al., 2014b
Transverse myelitis/Longitudinally extensive transverse myelitis	Relatively abrupt onset of motor, sensory, and sphincter disturbances due to an inflammatory demyelinating lesion/spinal lesion extending over at least three vertebral segments	Signs of inflammation in the CSF in most patients	Hyperintensity in T2-weighted images in spinal MRI	Renganathan et al., 1996; Solomon et al., 2000b; Leao et al., 2002; Kunishige et al., 2004; Seet et al., 2006; Chanthamat and Sathirapanya, 2010; Verma et al., 2011a; Larik et al., 2012; Singh et al., 2013; de Sousa et al., 2014; Weeratunga et al., 2014a; Fong et al., 2016; Mo et al., 2016; Mota et al., 2017
Acute disseminated encephalomyelitis	Acute inflammatory demyelinating disorder of the central nervous system, monophasic course, and multifocal white matter involvement that occur during or after dengue virus infection	Normal/ Inflammatory CSF	Extensive involvement of the white matter of the frontal, parietal, or temporal lobes; and lesions of basal ganglia, brainstem, cerebellum, corpus callosum, and periventricular regions	Yamamoto et al., 2002; Brito et al., 2007; Gera and George, 2010; Sundaram et al., 2010; Gupta et al., 2013; Viswanathan et al., 2016

*CSF, Cerebrospinal fluid; CT, computed tomography; MRI, magnetic resonance imaging*.

### Encephalitis

Encephalitis is considered a severe manifestation of dengue virus infection, and found in the three classical disease groups. Diagnosis of dengue encephalitis is based on criteria proposed by Soares and Marzia (2014).

However, normal CSF cellularity cannot exclude dengue encephalitis. According to Soares et al., dengue is the first reason for encephalitis with normal CSF cellularity in 75% of patients with viral meningitis and encephalitis in a dengue endemic region, followed by Herpes Simplex Virus 1 (HSV1) and mild encephalitis (Soares et al., 2011). Neuroimaging of dengue encephalitis yields divergent data, with normal findings in most cases (Baldaçara et al., 2013; Madi et al., 2014). In case of abnormal neuroimaging findings, magnetic resonance imaging (MRI) has advantages over cranial computed tomography (CT) in revealing cerebral lesions in dengue encephalitis. However, changes are usually non-specific (Garg et al., 2017). Decisive characterizations of MRI properties in dengue encephalitis remain undefined (Mathew et al., 2015). Treatment is nonspecific, with mostly symptomatic treatment provided. Most patients have good recovery.

### Encephalopathy

Encephalopathy caused by dengue fever can be reflected by reduced sensitivity, cognitive impairment, convulsions, and personality and behavior disorders, including acute mania, depression, emotional lability, anxiety, psychosis, and agoraphobia (Rittmannsberger et al., 2010; Baldaçara et al., 2013; Srivastava et al., 2013). In a review reporting cases of dengue fever associated with neurological disorders in 2012, encephalopathy was considered by far the most encountered complication (Oehler et al., 2012). Most encephalopathy cases occur in children of developing countries, and do not show CSF abnormalities (Angibaud et al., 2001). Dengue associated encephalopathy is generally very serious, with around 50% of the affected patients succumbing (Angibaud et al., 2001).

In the past, encephalopathy was considered to be exclusively associated with Dengue hemorrhagic fever/Dengue shock syndrome (DHF/DSS). Brain edema, anoxia, hemorrhage, intense hyponatraemia, liver or kidney failure, release of toxic substances, metabolic acidosis, and direct organ invasion are commonly reported precursors of encephalopathy in patients with serious DHF/DSS (Sumarmo et al., 1983; Nimmanitya et al., 1987; Srivastava et al., 1990; Hendarto and Hadinegoro, 1992; Thisyakorn and Thisyakorn, 1994; Sirivichayakul et al., 2000; Solbrig and Perng, 2015).

A substitution of alanine by a valine residue at position 173 of the envelope glycoprotein was reported in encephalopathy-associated dengue type 2 virus in 1996 (Sistayanarain et al., 1996). However, whether such mutation is directly involved in envelope-receptor interactions has not been clearly elucidated. Burst suppression, seizures, focal patterns, or epilepsia partialis continua can be observed on EEGs (electroencephalographs) of patients with encephalopathy (Kalita and Misra, 2006; Misra et al., 2006; Liou et al., 2008a,b).

### Meningitis

Dengue infection associated meningitis is rarely encountered (Soares et al., 2006). In the related literature, three reports about dengue meningitis are found (Soares et al., 2010, 2011; Mamdouh et al., 2013). Soares et al. described a 24-year-old woman with the symptoms of fever, headache, and nuchal rigidity, in whom diagnosis was confirmed by polymerase chain reaction (PCR) and positive results in CSF evaluation (Soares et al., 2010). Two additional cases of dengue meningitis were reported by Mamdouh in 2013. Both cases presented with fever, headache, neck rigidity, and low platelet count in a dengue endemic area. Diagnosis was confirmed by positive IgM in the CSF in the absence of the typical clinical spectrum of infection. CSF cellularity was normal in one case. Brain CT or MRI scan was normal. Dengue meningitis might cause migraine such as headaches with poor response to anti-migraine therapy and common analgesics. The two patients achieved full recovery after several months without any residual neurological deficit (Mamdouh et al., 2013).

### Stroke

Ischemic and hemorrhagic strokes have been reported in a few cases. Liou et al. reported a dengue fever patient who showed thrombocytopenia and ischemic stroke in 2008 (Liou et al., 2008a). In 2013, Rajesh Verma et al. reported a 68-year-old male patient presenting with moderate grade, continuous fever for 15 days; the patient also had sudden onset of weakness of the left half of his body as well as facial asymmetry, 10 days prior to hospital admission. Dengue infection was confirmed and brain MRI revealed acute infarction in the right parietal lobe. After 2 months of medication and physiotherapy, a partial improvement was observed in limb weakness (Verma et al., 2013). In cases with ischemic stroke, meningovasculitis, or a transient hypercoagulable state during dengue infection was postulated as the pathogenetic mechanism (Liou et al., 2008a; Verma et al., 2013).

A summary of intracerebral hemorrhage cases associated with dengue virus infection in various countries from 2001 to 2014 was reported by Vargas-Sánchez et al. (2014). In the above study, patient age ranged from 9 to 68 years, and there were 5 females and 7 males. Outcome was unspecified in one patient, while 7 and 5 patients recovered and died, respectively. Meanwhile, DENV2 was detected in 5 patients; one patient was infected with DENV3 and precision was not provided for the remaining seven. The involved regions included the pontine, basal gangliar, cerebellar, parietal, temporal, and frontal lobes of the brain (Vargas-Sánchez et al., 2014).

Current studies suggest that in dengue virus infection, cytokine overproduction results in immune-mediated endothelial cell damage (Seet and Lim, 2007; Basu and Chaturvedi, 2008). Hemorrhage may be caused by elevated vascular permeability, plasma leakage, and vasculitis due to dengue (Seet and Lim, 2007; Basu and Chaturvedi, 2008).

### Cerebellar syndrome

Cerebellar syndrome was mentioned by Weeratunga et al. (2014b). They described three patients diagnosed with dengue infection based on combined clinical and laboratory findings, fulfilling the WHO criteria in an endemic area. Within 2 weeks of diagnosis, they developed cerebellar symptoms. The manifestations of cerebellar syndrome include bilateral vertical and horizontal nystagmus, dysarthria, bilateral limb, and gait ataxia. Other causes of cerebellar dysfunction were excluded, and all cases were self-limiting. MR brain scans showed cerebellar T2 hyperintense lesions in one case, while the remaining two showed normal signals. The CSF in all cases had normal protein levels and cell count. All cases showed antibodies for dengue in blood and CSF samples. A low-grade inflammatory process was the proposed mechanism (Weeratunga et al., 2014b).

### Transversemyelitis(TM)/longitudinally extensive transverse myelitis (LETM)

The diagnosis of transversemyelitis (TM) is established by a somewhat sudden onset of sensorimotor and sphincter disturbances resulting from inflammatory demyelinating lesions (Scott et al., 2011). In longitudinally extensive transverse myelitis (LETM), a spinal lesion covers three or more vertebral segments (Wolf et al., 2012). According to Jacob and Weinshenker's opinions, TM can be broadly classified into four categories: (1) demyelination (monofocal clinical isolated syndrome) or multifocal demyelination [acute disseminated encephalomyelitis (ADEM) or multiple sclerosis]; (2) combined systemic connective tissue disease; (3) infection; (4) idiopathic illness (Jacob and Weinshenker, 2008).

Positive findings by spinal MRI are crucial in reaching the diagnosis of TM/LETM. Hyperintensity in T2-weighted signals found in spinal MRI scans support transverse myelitis diagnosis. In addition, inflammatory reactions in the CSF are found in most patients. TM/LETM in dengue is rarely encountered, and no more than 12 case reports are available (Renganathan et al., 1996; Leao et al., 2002; Kunishige et al., 2004; Seet et al., 2006; Chanthamat and Sathirapanya, 2010; Verma et al., 2011a; Larik et al., 2012; Singh et al., 2013; Weeratunga et al., 2014a; Fong et al., 2016; Mo et al., 2016; Mota et al., 2017).

However, the spectrum of TM could be broad, and more attention should be paid to atypical cases. For example, Mota reported a patient confirmed with acute TM without paraparesis following a dengue virus infection last year (Mota et al., 2017).

Treatment with intravenous steroids is useful, and individuals with parainfectious-dengue TM show satisfactory recovery (Fong et al., 2016). Fong et al. reported that a previously healthy 12-year-old girl with parainfectious-dengue TM and concomitant spinal epidural haematoma had good clinical recovery without surgical intervention after 6 months (Fong et al., 2016). It was shown that post-infection autoimmune reactions and direct infection are associated with transverse myelitis (Solomon et al., 2000b; Sindic et al., 2001; de Sousa et al., 2014; Mo et al., 2016).

### Acute disseminated encephalomyelitis (ADEM)

ADEM is characterized by an acute inflammatory demyelinating ailment affecting the CNS, a monophasic course, and multifocal white matter involvement which occurs during or after dengue virus infection (Puccioni-Sohler et al., 2013). PubMed and Scopus combinely reported only 7 cases prior to May 2017 (Yamamoto et al., 2002; Brito et al., 2007; Gera and George, 2010; Sundaram et al., 2010; Gupta et al., 2013; Viswanathan et al., 2016). ADEM onset occurs within a limited time (averaging 5.6 days) after initial dengue signs (Gupta et al., 2013). An abnormal CSF contributes to ADEM diagnosis, while a normal CSF cannotrule out its possibility (Viswanathan et al., 2016).

Few studies reporting radiologically confirmed dengue associated ADEM are available. MRIs show extensive involvement of the white matter of the frontal, parietal, or temporal lobe, basal ganglia, brainstem, cerebellum, corpus callosal, and periventricular lesions (Puccioni-Sohler et al., 2012, 2013; Gupta et al., 2013; Domingues and Kuster, 2014; Mudin, 2015; Viswanathan et al., 2016). Perivenous demyelination, macrophage influx, and perivascular infiltration of lymphocytes with hemorrhagic foci were reported after histological examination of such lesions (Sundaram et al., 2010).

Dengue related ADEM results from immune reactions (Gupta et al., 2013). In Murthy's study, its pathophysiology was considered a transient autoimmune reaction to myelin or unknown self-antigens (Murthy, 2010). There is no established treatment for ADEM, but the use of steroids is effective during its active phase (Carod-Artal et al., 2013; Gupta et al., 2013; Domingues and Kuster, 2014).

## Peripheral nervous system complications

Reviews indicated that peripheral nervous system signs comprise 5% of neurological symptoms in dengue fever. They usually occur later than CNS manifestations (Oehler et al., 2012). The associated peripheral syndromes mainly include Guillain-Barre syndrome, hypokalemic quadriparesis or plegia, mononeuritis multiplex, brachial plexitis, diaphragmatic paralysis, and myositis (Jha and Ansari, 2010; Sharma et al., 2011; Verma et al., 2011b; Gutch et al., 2012; Ratnayake et al., 2012; Jain et al., 2014; Table 2).

**Table 2:** Main peripheral nervous system complications associated with dengue infection.

**Complications**	**Main manifestion**	**CSF parameters**	**CT/MRI**	**References**
Guillain-Barre syndrome	Rapidly ascending paralysis, determined by an inflammatory demyelinating or axonal polyneuropathy	Protein-cytological dissociation	Normal	Paul et al., 1990; Sainte Foie et al., 1993; Chew et al., 1998; Esack et al., 1999; Gaultier et al., 2000; Santos et al., 2004; Sulekha et al., 2004; Kumar and Subhashini, 2005; Shah, 2007; Soares et al., 2008; Gupta P. et al., 2009; Oehler et al., 2012; Qureshi et al., 2012; Carod-Artal et al., 2013; Dupont-Rouzeyrol et al., 2014; Simon et al., 2016; Umapathi et al., 2016
Myositis	Mild asymmetrical weakness of the lower limbs to rapidly progressive severe limb and trunk weakness and even respiratory failure, myalgia, elevation of creatine phosphokinase	Normal	Normal	Malheiros et al., 1993; Kalita et al., 2005; Rajajee et al., 2005; Finsterer and Kongchan, 2006; Misra et al., 2006, 2012; Ahmad et al., 2007; Sangle et al., 2010; Paliwal et al., 2011; Pimentel et al., 2011; Siriyakorn and Insiripong, 2015
Hypokalemic paralysis	Acute neuromuscular weakness			Comi et al., 1985; Esack et al., 1999; Santos et al., 2004; Kalita et al., 2005; Jha and Ansari, 2010; Paliwal et al., 2011; Roy et al., 2011; Hira et al., 2012; Kayal et al., 2013; Jain et al., 2014, 2015; Maurya et al., 2016
Neuritis				
Brachial neuritis	Acute onset of severe unilateral shoulder pain, followed by flaccid paralysis of shoulder and paras-capular muscles a few days later			Verma et al., 2011b
Long thoracic nerve palsy	Sharp pain in the upper chest wall and shoulder, reduced the elevation of the involved arm			Chappuis et al., 2004
Phrenic neuropathy	Dyspnea and cough			Chien et al., 2008
Abducens nerve palsy	Binocular diplopia, convergent squint	Unremarkable		Shivanthan et al., 2012
Lateral rectus palsy	Diplopia, convergent squint, ocular movements disorder			Mishra et al., 2013
Peripheral facial palsy	Left/Right sided facial weakness with drooping of the mouth, drooling of saliva, and inability to close the left eyelid		Normal signal intensity within the brain parenchyma or in the visualized portions of the facial nerve	Patey et al., 1993; Peter et al., 2013

*CSF, Cerebrospinal fluid; CT, computed tomography; MRI, magnetic resonance imaging*.

### Guillain-Barré syndrome

Guillain-Barré syndrome (GBS) features a quickly rising paralysis, reflecting an inflammatory demyelinating or axonal polyneuropathy (Soares et al., 2008). In most cases, a protein-cytological dissociation in the CSF is indicative of GBS (Allos, 1998). Presentations of reduced conduction velocity, conduction blockage in motor nerves, extended distal latency, and prolongation or absence of F responses are often observed by electromyography in GBS patients (Soares et al., 2008).

GBS following dengue virus infection is uncommon (Meena et al., 2011; Qureshi et al., 2012; Chaudhary et al., 2014). In the literature, about 20 case reports of GBS associated with dengue infection are available (Paul et al., 1990; Sainte Foie et al., 1993; Esack et al., 1999; Santos et al., 2004; Sulekha et al., 2004; Kumar and Subhashini, 2005; Soares et al., 2008; Oehler et al., 2012; Carod-Artal et al., 2013; Dupont-Rouzeyrol et al., 2014; Simon et al., 2016). Most cases were pediatric patients, and only few adults were involved (Chew et al., 1998; Gupta P. et al., 2009; Qureshi et al., 2012).

Neurological signs develop at 1–19 days after the onset of dengue. Simon et al. found GBS occurs early in DF with an unusual progression; in their report, average time elapsed from fever to neurological signs was 2 days, ranging between 1 and 3 days (Simon et al., 2016). A patient with Miller Fisher syndrome caused by dengue has been described (Gaultier et al., 2000). Umapat et al. reported that asymptomatic dengue infection may also trigger Guillain-Barré syndrome, and found four GBS cases after dengue diagnosis, including 1 and 3 with acute motor axonal neuropathy and acute inflammatory demyelinating polyneuropathy, respectively (Umapathi et al., 2016).

The exact mechanism of dengue associated GBS remains unclear. It is considered a neurological disease modulated by immunocytes (Chew et al., 1998; Gupta P. et al., 2009). Pro-inflammatory substances such as tumor necrosis factor, interleukins, and the complement may play important roles in GBS pathogenesis (Shah, 2007). Immune reactions induced by dengue virus infection might involve peripheral nerve constituents; for example, myelin or axon may be targeted by the immune response (Carod-Artal et al., 2013).

Plasma exchange seems to be more effective than supportive treatments in the treatment of GBS in randomized clinical trials (Comi et al., 1985; Qureshi et al., 2012). Immunoglobulins administered intravenously show similar effectiveness to plasma exchange, and might be more advantageous (Chew et al., 1998; Gupta P. et al., 2009; Qureshi et al., 2012). Corticosteroids alone do not make a difference, and there is not enough data supporting their usefulness (Comi et al., 1985; Qureshi et al., 2012). Other treatment options, e.g., CSF filtration, are still being assessed (Qureshi et al., 2012).

### Myositis

Dengue myositis diagnosis is based on clinical manifestations of dengue virus infection, positive serum IgM for the dengue virus, high creatine phosphokinase levels, normal CSF, and exclusion of other causes (Paliwal et al., 2011). The clinical spectrum is broad, from mild asymmetrical weakness of lower extremities to sudden progressive severe limb and trunk weakness, and even lung failure (Paliwal et al., 2011). However, muscle weakness is distinctly uncommon in dengue virus infection (Malheiros et al., 1993; Sangle et al., 2010). Doctors should pay more attention to patients suffering from early pulmonary impairment, elevated creatine phosphokinase amounts, and serious myalgia (Paliwal et al., 2011).

The pathogenesis of myositis remains unclear. The proposed mechanisms comprise direct muscular invasion by the virus and immunity associated destruction of muscle fibers, particularly by tumor necrosis factor (Malheiros et al., 1993; Paliwal et al., 2011). Dengue myositis is reflected by perivascular infiltration of mononuclear cells, mitochondrial proliferation, fat accumulation, nuclear centralization, fiber-type grouping, and/or myonecrosis foci on muscle biopsy (Malheiros et al., 1993; Misra et al., 2012).

Normal motor unit potentials are of reduced duration and amplitude, and polyphasic on electromyographic examination. Fibrillations, sharp waves, and complex repetitive discharges do not occur (Kalita et al., 2005; Misra et al., 2012).

However, dengue myositis is considered a relatively benign and self-limiting disease in pediatric reports (Rajajee et al., 2005; Misra et al., 2006; Ahmad et al., 2007; Pimentel et al., 2011). In adult patients, dengue myositis is often more severe, even leading to severe rhabdomyolysis (Kalita et al., 2005; Finsterer and Kongchan, 2006; Siriyakorn and Insiripong, 2015). Sangle et al. reported a 16-year-old girl with dengue shock syndrome showing myositis and myocarditis. Symptomatic treatment and rehabilitation were provided, and she had recovered well at discharge after 1 month of hospitalization (Sangle et al., 2010). Finsterer et al. found dengue can cause persistent and serious myositis, which is resolved after administration of corticosteroids (Finsterer and Kongchan, 2006).

### Hypokalemic paralysis

Dengue-associated hypokalemic paralysis was reported by several authors, but most studies were based on isolated case reports and short series (Esack et al., 1999; Santos et al., 2004; Kalita et al., 2005; Jha and Ansari, 2010; Roy et al., 2011; Jain et al., 2014; Maurya et al., 2016). Maurya and colleagues assessed 12 dengue patients with hypokalemic paralysis, who had complete and rapid recovery after potassium supplementation (Maurya et al., 2016).

It has been suggested that elevated creatine phosphokinase amounts result from vasoconstriction and muscular ischemia caused by hypokalemia (Comi et al., 1985). In a study of 12 patients with acute neuromuscular weakness in a dengue epidemic, 10 individuals showed hypokalemia, while Guillain-Barré syndrome and myositis were found in one patient each. Of the latter 2 patients, the one with myositis showed slow improvement and normal serum potassium levels, and the other with Guillain-Barré syndrome recovered within 6 weeks (Hira et al., 2012). In a retrospective study of 7 patients with dengue myositis who had generalized weakness, a mild serum creatin phosphokinase (CPK) elevation was noted in 3 individuals with hypokalemia, whereas three cases with fulminant presentation and respiratory muscle involvement had markedly elevated CPK levels (16,590-117,200 U/L) with normal potassium amounts. All patients completely recovered within 4 weeks (Paliwal et al., 2011).

The severity of weakness may not correlate with potassium levels in Maurya's report (Maurya et al., 2016). Hypokalemia does not always lead to paralysis. In 1,342 patients with dengue fever assessed in China, hypokalemia occurred in up to 28% individuals but weakness was not mentioned (Ying et al., 2007).

Hypokalemic paralysis differs from dengue myositis and idiopathic hypokalemic paralysis according to clinical, biochemical features, and consequences (Maurya et al., 2016). It represents a systemic complication of dengue fever (Murthy, 2010; Jain et al., 2014). The pathogenesis of hypokalemic paralysis in dengue remains obscure. A probable explanation for hypokalemia in dengue is serum potassium redistribution in cells and transient kidney tubular dysfunction increasing urinary potassium elimination (Jha and Ansari, 2010; Jain et al., 2015; Maurya et al., 2016). In addition, infection-related stress may lead to catecholamine or insulin release, which results in an intracellular shift of potassium (Jha and Ansari, 2010; Hira et al., 2012). Supplementation of potassium can achieve satisfactory recovery in patients with hypokalemia paralysis (Jha and Ansari, 2010; Kayal et al., 2013). Jain et al. reported a 30-year-old man with dengue and hypokalemic paralysis alongside hypomagnesemia. Weakness persisted after potassium supplementation, while low serum magnesium levels were detected. However, he completely recovered within 48–72 h, with a normalization of serum potassium and magnesium levels. The possible mechanism may be that muscle Na+ and K+-ATPase activity is inhibited by hypomagnesemia, leading to a reduced ion influx into muscle fibers and secondary kaliuresis (Jain et al., 2015).

### Neuritis

Neuritis after dengue infection is believed to rarely occur. However, recent studies have reported more and more dengue fever patients showing uncommon neurological signs. Dengue-associated neuritis, such as brachial neuritis, long thoracic nerve palsy, phrenic nerve palsy, abducens nervepalsy, and peripheral facial palsy have been reported in different areas of the world (Patey et al., 1993; Chappuis et al., 2004; Chien et al., 2008; Verma et al., 2011b; Shivanthan et al., 2012; Mishra et al., 2013; Peter et al., 2013).

Its diagnosis is established, after other reasons for neuritis, such as tumor and demyelinating diseases, infections, trauma, and stroke, are accordingly excluded. Dengue-associated nerve palsy is mostly managed by supportive treatment. Some cases improve even without specific treatment, e.g., with steroids or intravenous immunoglobulins (Peter et al., 2013; Biswas and Pal, 2014), while others have a good response to steroids (Verma et al., 2011b).

The pathogenesis of dengue-associated neuritis is likely related to immune reactions, although the mechanisms remain largely unclear (Carod-Artal et al., 2013). Most patients achieve a remarkable recovery.

## Ophthalmic complications

Ophthalmic complications in dengue fever were previously considered rare events, but more cases have been reported (Carod-Artal et al., 2013). Multiple studies identify maculopathy as the most frequent neuro-ocular sign; less commonly encountered complications are optic neuropathy, retina vasculopathy, and cranial nerve palsy (Yip et al., 2012). Kapoor et al. retrospectively assessed 134 dengue fever patients, among whom up to 40% had ocular manifestations (Kapoor et al., 2006). Ocular complications involve: (1) the anterior segment of the eye, e.g., subconjunctival hemorrhage, uveitis, or a shallow anterior chamber (Cruz-Villegas et al., 2003; Pierre Filho Pde et al., 2008); (2) the posterior segment of the eye, e.g., maculopathy, macular edema, optic neuropathy, or vitreous hemorrhage (Nainiwal et al., 2005; Bacsal et al., 2007; Chang et al., 2007; Tan et al., 2007; Kanungo et al., 2008; Loh et al., 2008; Quek et al., 2009). Presentation of dengue-related ocular signs and symptoms often corresponds to thrombocytopenia (Chan et al., 2006; Teoh et al., 2006). However, some complications, e.g., uveitis, can occur 3–5 months after dengue infection (Gupta A. et al., 2009). The majority of individuals with such complications are spontaneously relieved. Systemic steroids and occasional immunoglobulins are provided to patients with severe vision loss. The prognosis of dengue-related ophthalmic complications is favorable. Almost all patients become normal or improve in vision (Yip et al., 2012).

The mechanisms underlying dengue infection-related ocular signs remain unclear, but could involve immune processes with possible association with dengue serotyping (Chan et al., 2006; Bacsal et al., 2007; Su et al., 2007; Yip et al., 2012).

## Neurological complications in children

Dengue causes high morbidity and mortality in children living in tropical and subtropical areas of the world. Around 95% of patients with serious disease are below 15 years old (Bhattacharya et al., 2013). All four dengue virus serotypes can be detected in infectious children, and clinical symptoms range from mild fever to fatal dengue shock syndrome (Verhagen and de Groot, 2014).

National surveillance in Asia showed that individuals below 1 year old and those between 4 and 9 years of age are most likely to develop severe dengue infection (Kongsomboon et al., 2004; Huy et al., 2010). It is worth noting that compared with older children, infants often show a higher frequency of plasma leakage and shock in dengue (Nguyen et al., 2004; Hammond et al., 2005). Therefore, the management of infants with dengue infection is important because they sometimes present with unusual manifestations, and early diagnosis is very challenging (Kalayanarooj and Nimmannitya, 2003). Whether severe dengue disease occurs or not largely depends on factors such as virus properties, host immunity, age, and genetic makeup. Female children may be associated with severe dengue (Anders et al., 2011). Prognosis of dengue hemorrhagic fever and dengue shock syndrome is affected by prevention, early detection, and timely therapy; mortality ranges between 2.5 and 5.0% (Alejandria, 2009). In case of shock, mortality can approach 12–44% (Rigau-Pérez et al., 1998). Patients usually have satisfactory recovery after optimal fluid/electrolyte supplementation.

Neurological complications recorded in pediatrics include ADEM (Kamath and Ranjit, 2006), hepatic encephalopathy (Kamath and Ranjit, 2006), acute childhood myositis (Ahmad et al., 2007), hemiconvulsion-hemiplegia-epilepsy (Gastaut et al., 1960; Saini et al., 2017), parkinsonism (Fong et al., 2014), ischemic stroke due to dengue vasculitis (Nanda et al., 2014), sub-arachnoid hemorrhage (Kamath and Ranjit, 2006), and transverse myelitis (Fong et al., 2016). Most cases achieve satisfactory recovery after timely treatment (Kankirawatana et al., 2000; Cam et al., 2001; Kamath and Ranjit, 2006). Children intervened late are harder to resuscitate (Kamath and Ranjit, 2006).

Hospitalization may not be necessary for children with mild dengue infection (Nguyen et al., 2004; Hammond et al., 2005). There are no specific therapeutic agents for dengue, but fluid replacement is immediately required in pediatric cases with haemorrhagic fever or shock syndrome, to expand the plasma volume. Crystalloids are as potent as colloids in children with moderately severe and severe dengue shock syndrome (Alejandria, 2009). For children with suspected DHF, attentive clinical monitoring and supportive care are critical measures for reducing fatality rates (Tantawichien, 2012). Preventive transfusions are not recommended (Tantawichien, 2012), and steroid administration is not considered a beneficial option in dengue shock syndrome (Smart and Safitri, 2009).

## Therapy and prevention

Currently, no definite effective antiviral agents are available for dengue infection treatment. General supportive therapy prevails, emphasizing on intense hematological monitoring, fluid-replacement, and/or blood transfusion if needed. Non-steroidal anti-inflammatory drugs may worsen gastritis or cause bleeding.

A safe and efficacious dengue vaccine is considered a great hope for preventing and controlling this disease. Currently, prevention is only achieved by vector control. However, several vaccine preparations are under investigation (Simmons et al., 2012). Notably, it is especially important for children to avoid *Aedes* mosquito bites in dengue-endemic regions (Elling et al., 2013).

## Comparsion with Japanese encephalitis virus

Dengue is considered a non-neurotropic virus. However, neurotropism and neuro-invasion in dengue have been reported (Chardboonchart et al., 1990; Despres et al., 1998; Solomon et al., 2000b; Cam et al., 2001). DF is usually self-limiting, and death is rather uncommon (Tantawichien, 2012). *In vitro* experiments in 1998 indicated DENV directly infects neurons, which results in permanent damage (Despres et al., 1998). In 2000, Jan and colleages demonstrated that phospholipase A2 (PLA2) activation, cytochrome C release from the mitochondria, superoxide anion production, and nuclear factor-kB translocation could lead to neuronal apoptosis (Jan et al., 2000).

The major pathogenetic mechanisms include endothelial cell disfunction and development of coagulation disorders. Therefore, the clinical signs of dengue fever mostly comprise endothelial cell damage, enhanced vascular permeability, and increased plasma leakage (Halstead and Cohen, 2015). The dengue virus shows tropisms mainly for monocytes, macrophages, and dendritic cells (Jessie et al., 2004; Martina et al., 2009).

Though belonging to the same flavivirus group, Japanese encephalitis virus (JEV) is a proven neurotropic virus, and mainly targets neuronal cells (Kimura-Kuroda et al., 1993). Nearly 75% of symptomatic patients show manifestations of encephalitis, which often leads to various neurological complications or patient fatality (Lee et al., 2012; Sarkari et al., 2012a,b). JEV infection is considered a leading cause of pediatric encephalitis (Thongtan et al., 2012).

Little is known about the mechanism by which the virus spreads to the CNS as well as its brain tropism (Myint et al., 2007). Ghosh Roy et al. assumed that direct spread to the CNS occurs when mosquitos directly bite into blood vessels (Ghosh Roy et al., 2014).

Apoptosis induced by JEV involves the following three related mechanisms: direct neuron infection, infection of other CNS cells (e.g., microglia and astrocytes), and inflammatory reactions (Chen et al., 2004; Raung et al., 2007; Das and Basu, 2008; Swarup et al., 2008). As a result, CNS cell infection by JEV causes considerable neuronal apoptosis (Chen et al., 2004; German et al., 2006; Ghoshal et al., 2007). Serious vascular congestion, cerebral edema, neuronal death, astrocyte activation, and microglial proliferation are found in fatal cases of JEV infection (Misra and Kalita, 2010).

Therefore, it is relatively easy to understand why up to one-third of JE cases hospitalized die while about one-half of all survivors show permanent neurological sequelae, even individuals with apparent recovery (Kumar et al., 1993; Solomon et al., 2000a). However, most patients even with neurological manifestations in dengue infection have an unexpected good recovery with no obvious sequelae.

Dengue is gradually becoming a main public health problem worldwide. A growing number of related studies demands increased awareness and understanding of the neurological complications of dengue infection. Physicians, especially neurologists, will continue to play important roles in its diagnosis and treatment. Pathogenesis, detection, antivirals, vaccines, environmental risk reduction, and vector control still deserve further studies.

## Author contributions

GL: Designed the study, collected data, wrote the manuscript. YL: Designed the study, reviewed the manuscript. XL: Designed the study, reviewed the manuscript. ZN: Collected data and edited the manuscript.

### Conflict of interest statement

The authors declare that the research was conducted in the absence of any commercial or financial relationships that could be construed as a potential conflict of interest.
